# Knowledge and Awareness of Glaucoma: A Comparative Study Among Rural and Urban Patients in Bangladesh

**DOI:** 10.7759/cureus.102239

**Published:** 2026-01-24

**Authors:** Habiba Sultana, Khairul Islam, Md Sajidul Huq, Shajeda Azizi, Md Rezwanul Hasan

**Affiliations:** 1 Glaucoma, Deep Eye Care Foundation, Rangpur, BGD; 2 Cataract, Deep Eye Care Foundation, Rangpur, BGD; 3 Public Health, Deep Eye Care Foundation, Rangpur, BGD; 4 Vitreo-Retina, Deep Eye Care Foundation, Rangpur, BGD

**Keywords:** awareness, blindness, glaucoma, knowledge, rural, urban

## Abstract

Background: Glaucoma is a leading cause of irreversible blindness worldwide and often progresses silently until significant vision loss occurs. Early detection and timely treatment are crucial to preventing visual impairment; however, these efforts largely depend on public awareness. Assessing glaucoma-related knowledge, particularly among underserved populations, is therefore essential for developing effective prevention and control strategies. This study aimed to assess and compare the level of knowledge and awareness of glaucoma among rural and urban patients.

Methodology: This comparative study included a total of 706 respondents, with 353 participants each from urban and rural areas. Participants were recruited at the Deep Eye Care Foundation in Rangpur, Bangladesh, between April and September 2023. Ethical approval was obtained from the Ethical Review Committee of the Deep Eye Care Institute, and written informed consent was secured from all participants aged 18 years and older. A bilingual questionnaire was used to collect demographic data and assess knowledge and awareness of glaucoma. Data were analyzed using IBM SPSS Statistics for Windows, Version 26.0 (IBM Corp., Armonk, NY, USA), with a p-value < 0.05 considered statistically significant.

Results: The mean age was 39.31 ± 14.1 years for urban participants and 52.01 ± 14.2 years for rural participants. Educational attainment differed significantly, with 81 (22.9%) urban participants holding a graduate degree compared with 159 (45.0%) rural respondents having no formal education. Economic disparities were also evident: 29 (8.2%) urban families earned more than BDT 50,000 per month, whereas 186 (52.7%) rural participants earned less than BDT 10,000. Overall awareness of glaucoma was low, with only 147 (20.8%) participants familiar with the term, slightly higher among urban participants (80, 22.7%) than rural participants (67, 19%). A large proportion of both urban (288, 81.6%) and rural (287, 81.3%) respondents were unaware of or unsure about glaucoma. Rural participants reported a higher prevalence of glaucoma history (43, 64.2% vs. 11, 13.3%) and family history (36, 53.7% vs. 6, 7.4%). Medical treatment was the most commonly reported management approach, particularly among rural participants (35, 81.4% vs. 6, 54.5% urban). Knowledge of glaucoma was significantly better among urban participants, with 26 (32.5%) correctly identifying optic nerve involvement compared with 8 (11.9%) rural participants. Similarly, 41 (51.2%) urban respondents recognized increasing age as a risk factor, versus 11 (16.4%) rural respondents. A significant association was observed between residence and knowledge level (p < 0.001), with excellent knowledge reported in 25 (31.3%) urban participants compared with 6 (9.0%) rural participants. Overall, only 22 (15.0%) participants demonstrated good knowledge of glaucoma.

Conclusion: This study demonstrates a marked disparity in glaucoma awareness and knowledge between rural and urban populations, with rural communities showing substantially lower levels of understanding. These findings highlight the urgent need for targeted, community-based eye health education and screening programs, particularly in rural areas, to enhance early detection, encourage timely treatment, and reduce preventable blindness due to glaucoma.

## Introduction

Glaucoma is a chronic, progressive optic neuropathy characterized by specific alterations in the optic disc and visual field loss, frequently associated with increased intraocular pressure (IOP) [1]. The gradient across the lamina cribrosa is significantly increased by elevated IOP and low perfusion pressure, resulting in papillary hypoperfusion. This process leads to structural changes and remodeling of the lamina cribrosa, as well as impaired axonal transport in the optic nerve fiber [2]. It is the primary cause of irreversible blindness worldwide and is the second most common cause of blindness globally, following cataracts. Unfortunately, due to its insidious nature, it is often diagnosed in advanced stages, leading to severe ocular damage [3]. Early intervention can help avert permanent vision loss, but a general lack of awareness and inadequate screening mechanisms contribute to delayed diagnoses, even among the educated population [4].

The global glaucoma prevalence among individuals aged 40-80 years is estimated at 3.54%. Primary open-angle glaucoma (POAG) is most prevalent in Africa, while primary angle-closure glaucoma (PACG) is more common in Asia. In 2013, an estimated 64.3 million people aged 40-80 years were affected by glaucoma worldwide. This number increased to 76.0 million in 2020 and is projected to reach 111.8 million by 2040 [5]. In Bangladesh, the prevalence of confirmed glaucoma among those aged 40 years and older ranges from 2.1% [6] to 3.2%, with an additional 10.1% categorized as glaucoma suspects [7].

Several studies have suggested that a significant number of glaucoma cases, spanning from 50% to 90%, remain undiagnosed [8]. Lack of awareness is frequently the primary cause of delayed glaucoma diagnosis, which greatly raises the risk of blindness from glaucoma [3,4]. Awareness is shaped by several socioeconomic variables, such as educational attainment, family history, availability of recreational sources, and outreach by public or non-governmental health education initiatives [8]. Limited awareness not only delays diagnosis but also deters people from utilizing essential eye care services [9]. 

There is no denying that glaucoma has significantly impacted family finances [10]. In low- and middle-income countries, the economic toll of blindness is particularly severe due to productivity losses and caregiving costs, which constrain national resources [11]. Financial hardship often undermines treatment adherence, and as the disease advances, patients require more frequent tests, medications, and clinical visits, driving up expenses [12]. The cornerstone of glaucoma treatment is IOP reduction, which is the only proven method to halt disease progression. Determining an individual’s target IOP involves considering several factors, including baseline pressure, age, optic nerve condition, and risk of progression. The American Academy of Ophthalmology recommends an initial 25% IOP reduction from baseline for managing POAG [13]. Surgical approaches, such as laser trabeculoplasty or trabeculectomy, are considered when medication fails to control IOP [14].

Major risk factors for glaucoma include increasing age, high IOP, a family history of the condition, and ethnic background [1]. Unfortunately, glaucoma is frequently detected at an advanced stage, resulting in considerable vision loss [15]. For this reason, assessing public knowledge and awareness is essential to formulate effective educational and communication strategies. Improving awareness can promote routine eye exams and help mitigate the financial strain associated with this condition [4,9]. This study was undertaken to assess the knowledge and awareness of glaucoma among rural and urban populations, to combat this sight-threatening disease.

## Materials and methods

The study was conducted from April to September 2023. A total of 706 participants aged 18 years or older were included from rural and urban areas in the Rangpur district. The rural patients were selected from rural eye camps of the Deep Eye Care Foundation in Rangpur, Bangladesh. The urban population was selected from the general outpatient department of the Deep Eye Care Foundation. Individuals who consented to participate were briefed about the purpose of the study and were provided with a written consent form to sign. The study was conducted by doctors, optometrists, and refractionists who were trained on the questionnaire and had extensive knowledge of glaucoma. Inclusion criteria included patients over 18 years of age who provided informed written consent. Patients who were severely ill, unwilling to participate, under the age of 18, or mentally unstable were excluded from the study.

Sample size

The sample size for this study was calculated using the following formula:

$n= \Bigg([P1(100-P1)+ P2(100-P2)]/(P1-P2)^2 \Bigg) (Z&alpha;+Z&beta;)^2$

where *n* represents the sample size, *Zα* denotes the Z-value of the standard normal deviation at a given level of significance (1.28 at 20% level of significance), *Zβ* states the standard normal deviation at a given power (typically set at 0.84 for an 80% power), *P1* indicates the assumed prevalence of the awareness level in the urban group based on a previous study (25.94%), and *P2* represents the estimated prevalence of the awareness level in the rural group from the same study (19.9%) [16].

By substituting these values into the formula, the initial estimated sample size was approximately 433 participants for each group (urban and rural). However, due to the unavailability of eligible participants, the targeted sample size could not be achieved. Consequently, a total of 706 participants were ultimately enrolled in the study, with 353 participants included in each group.

Development of the questionnaire

The survey questionnaire was developed after reviewing relevant literature and previously published studies on glaucoma awareness and knowledge [15,17-19]. Participants were asked to answer a pretested, semi-structured questionnaire to assess their awareness and knowledge of glaucoma. It was designed to collect information on three main areas: participants’ sociodemographic characteristics, awareness of glaucoma, and knowledge of the disease among those who were aware. The questionnaire was initially drafted in English and then translated into the local language, with back-translation performed to ensure accuracy and clarity.

Testing of the questionnaire

A pretest was conducted on a small sample of participants who were not included in the final study to assess the clarity of the questions, ease of understanding, and the practical feasibility of administering the questionnaire, including the time required and the smoothness of the interview process. Based on the feedback from the pretest, minor modifications were made to improve readability and overall administration.

Validation of the questionnaire

Content validity was established through expert review by ophthalmologists and public health specialists to ensure that all questions were relevant, clear, and comprehensive. Reliability was ensured by standardizing the administration procedures, which included providing consistent instructions to participants, maintaining the same order of questions, using a uniform method of delivery (face-to-face interviews), and addressing participants’ queries in a predefined manner. Scoring methods were also standardized to ensure consistency in evaluating responses.

Assessment of awareness and knowledge

The respondents who had heard of glaucoma were considered aware of the disease, and their knowledge was assessed based on their understanding of it. Participants who were unable to answer any questions were deemed not to know about glaucoma. Following the collection of demographic data, participants were asked awareness-related questions, including inquiries about medical history (e.g., history of diabetes mellitus (DM) and hypertension (HTN)), history of eye examinations, family history of glaucoma, and their sources of information. Awareness was assessed by asking participants, “Have you ever heard of the term ‘Glaucoma’?” [15].

Knowledge details were obtained only from participants who were aware of glaucoma [18]. Knowledge was assessed by questioning participants about the altered anatomical site, different types, clinical presentation, risk factors, association with high IOP and visual field, and treatment options for glaucoma. A Likert scale was used to measure the respondents’ knowledge and analyze their responses, where 1 = Yes, 2 = No, and 3 = I don’t know [15]. For each question, a correct answer was given a +1 score, while a wrong answer received no score. The sum of the scores from all 10 knowledge-related questions provided the final score for each participant. The knowledge level was then divided into three grades: poor, good, and excellent. Participants’ knowledge was categorized as “Excellent” if they achieved more than 75% of the total score, “Good” if they scored between 50% and 75%, and “Poor” if they scored less than 50% [17].

Data handling and analysis

The obtained data were entered into a Microsoft Excel (Microsoft Corp., Redmond, WA, USA) spreadsheet and analyzed using the IBM SPSS Statistics for Windows, Version 26.0 (Released Year; IBM Corp., Armonk, NY, USA). The chi-square test was applied, and a p-value less than 0.05 was considered statistically significant.

Ethical approval

Ethical approval was obtained from the Institutional Review Board (IRB) of the Deep Eye Care Foundation, Rangpur, Bangladesh (Ref. No: IRB/DECF/2023/R12).

## Results

Table 1 presents the sociodemographic characteristics of the participants. The mean age of urban and rural participants was 39.31 ± 14.1 and 52.01 ± 14.2 years, respectively. In urban areas, most participants were aged 41-60 years (41.6%), followed by 21-40 years (40.5%). In rural areas, the largest group was also 41-60 years (42.8%), followed by ≥61 years (30.9%), compared to 6.5% in urban areas. Female participants predominated in urban areas (55.8%), while males were more common in rural areas (56.9%). A stark contrast was observed in educational attainment. Urban participants were more educated, with 22.9% being graduates and 12.5% postgraduates, while rural respondents had a high rate of no formal education (45%). Regarding occupation, urban areas had more homemakers (31.7%) and students (16.4%), whereas rural areas had higher proportions of homemakers (26.1%), farmers (25.8%), and business workers (21.8%). Urban families generally had higher incomes, with 8.2% earning over BDT 50,000 compared to only 1.1% in rural areas. More than half of rural participants (52.7%) earned less than BDT 10,000 monthly, reflecting significant economic disparities.

**Table 1:** Sociodemographic characteristics of the participants (N = 706)

Parameters	Urban (n = 353)	Rural (n = 353)
Age (years)		
≤20	40 (11.3)	5 (1.4)
21-40	143 (40.5)	88 (24.9)
41-60	147 (41.6)	151 (42.8)
≥61	23 (6.5)	109 (30.9)
Mean ± SD	39.31 ± 14.1	52.01 ± 14.2
Gender		
Male	156 (44.2)	201 (56.9)
Female	197 (55.8)	152 (43.1)
Education		
No formal education	40 (11.3)	159 (45)
Primary education	49 (13.9)	96 (27.2)
Secondary education	70 (19.8)	51 (14.4)
Higher secondary	69 (19.5)	24 (6.8)
Graduate	81 (22.9)	18 (5.1)
Post-graduate	44 (12.5)	5 (1.4)
Occupation		
Homemaker	112 (31.7)	92 (26.1)
Business	43 (12.2)	77 (21.8)
Farmer	12 (3.4)	91 (25.8)
Service holder	56 (15.9)	29 (8.2)
Student	58 (16.4)	9 (2.5)
Unemployed	21 (5.9)	31 (8.8)
Retired	12 (3.4)	9 (2.5)
Day labor	9 (2.5)	12(3.4)
Others	30 (8.5)	3 (0.8)
Monthly family income (BDT)		
<10,000	124 (35.1)	186 (52.7)
10,000-20,000	44 (12.5)	97 (27.5)
20,001-30,000	92 (26.1)	53 (15)
30,001-40,000	41 (11.6)	9 (2.5)
40,001-50,000	23 (6.5)	4 (1.1)
>50,000	29 (8.2)	4 (1.1)

**Figure 1:**
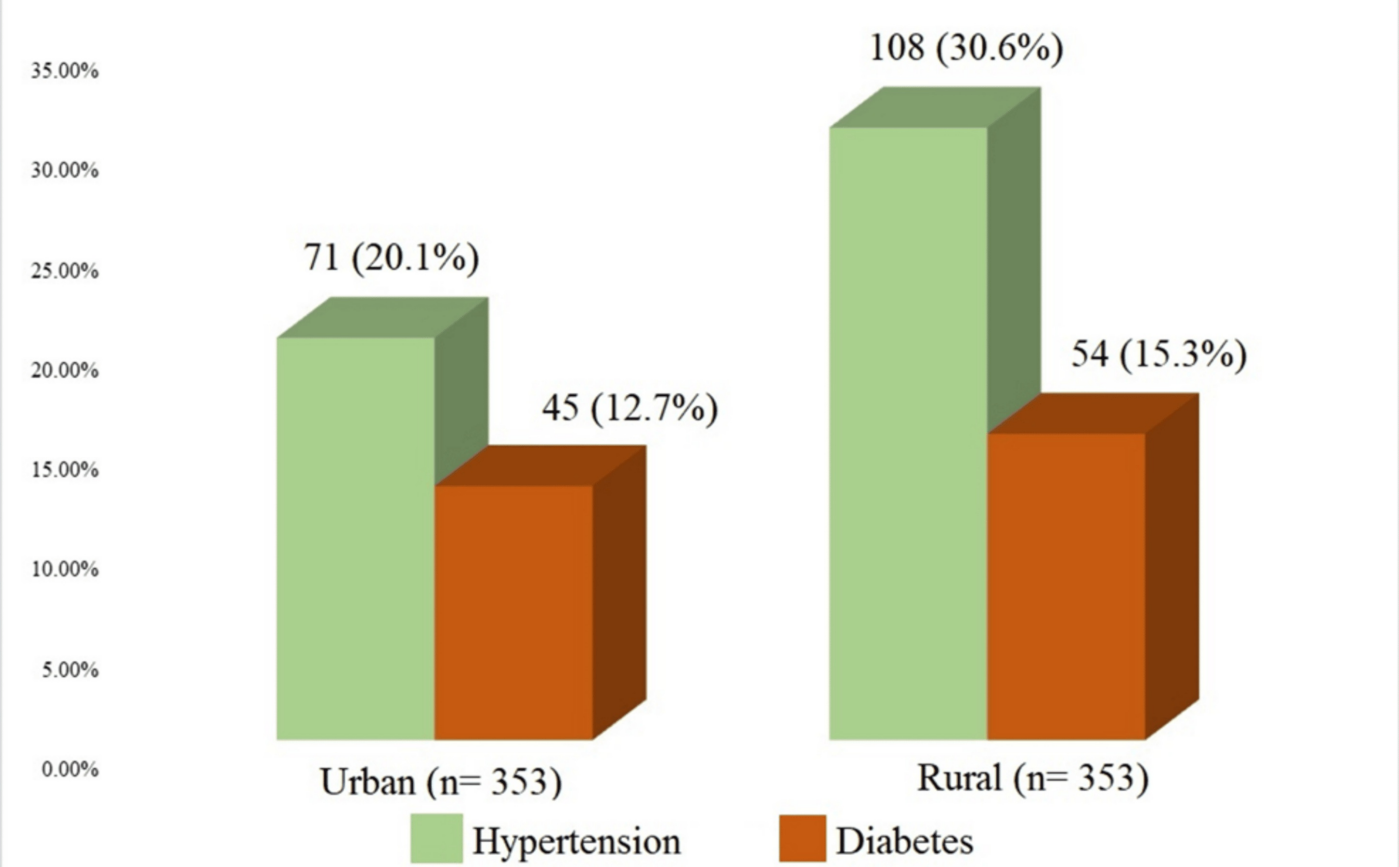
History of comorbidities

Figure 1 shows that both HTN (30.6%) and diabetes (15.3%) were more prevalent among rural participants than urban participants, with hypertension being the more common comorbidity in both groups.

Table 2 presents glaucoma awareness among participants. Rural participants were more likely to have had an eye exam (78.2%) compared to urban participants (64.9%) (p < 0.001). In urban areas, most eye exams occurred more than 12 months ago (51.1%), while in rural areas, most eye exams (56.9%) were recent (within 12 months), with no significant difference between urban and rural participants. Both urban (86%) and rural (49.6%) participants primarily visited private hospitals or clinics, whereas rural participants often relied on government facilities (33.7%) and outreach services (16.7%) (p < 0.001). Only 20.8% of participants had heard of the term “glaucoma,” with urban participants slightly more aware than rural participants (22.7% vs. 19%, p < 0.001). Just 17.3% recognized it as an eye disease. Alarmingly, 81.6% of urban and 81.3% of rural respondents either did not know or were unsure (p < 0.001). In rural areas, family members (47.8%) were the primary source of information, whereas urban participants relied more on relatives/friends (33.8%) and healthcare professionals (27.5%). A history of glaucoma was more common among rural respondents (64.2% vs. 13.3%, p < 0.001), as was a family history (53.7% vs. 7.4%, p < 0.001), with mothers being the most frequently reported affected family member (47.6%). Among participants with glaucoma, medical treatment was the most common approach (75.9%), more prevalent among rural participants (81.4%) than urban participants (54.5%) (p = 0.005). Very few reported laser or surgical treatments.

**Table 2:** Patients' awareness-related questions (N = 706) Values are expressed as frequency and percentage. The statistical relationship was assessed using the chi-square test.

Questions	Parameters	Urban (n = 353)	Rural (n = 353)	p-value	Chi-square value	Total, n (%)
History of eye examination	Yes	229 (64.9)	276 (78.2)	<0.001	15.364	505 (71.5)
	No	124 (35.1)	77 (21.8)			201 (28.5)
If yes, time of examination	Within 12 months	112 (48.9)	157 (56.9)	0.074	3.198	269 (53.3)
	Before 12 months	117 (51.1)	119 (43.1)			236 (46.7)
If yes, place of examination	Government hospital/clinic	24 (10.5)	93 (33.7)	<0.001	74.482	117 (23.2)
	Private hospital/clinic	197 (86)	137 (49.6)			334 (66.1)
	Outreach	8(3.5)	46(16.7)			54 (10.7)
Have you ever heard of the term glaucoma before?	Yes	80 (22.7)	67 (19)	<0.001	22.063	147 (20.8)
	No	249 (70.5)	284 (80.5)			533 (75.5)
	Don’t know	24 (6.8)	2 (0.6)			26 (3.7)
Do you know that glaucoma is an eye disease?	Yes	58 (16.4)	64 (18.1)	<0.001	549.017	122 (17.3)
	No	7 (2)	287 (81.3)			294 (41.6)
	Don’t know	288 (81.6)	2 (0.6)			290 (41.1)
Source of information	Family member	12 (15)	32 (47.8)	<0.001	20.858	44 (29.9)
	Relatives/friends	27 (33.8)	17 (25.4)			44 (29.9)
	Health professional	22 (27.5)	13 (19.4)			35 (23.8)
	Mass media	19 (23.5)	5 (7.5)			24 (16.3)
Do you have a history of glaucoma?	Yes	11 (13.3)	43 (64.2)	<0.001	47.426	54 (36)
	No	47(56.6)	8 (11.9)			55 (36.7)
	Don’t know	25 (30.1)	16 (23.9)			41 (27.3)
If yes, types of treatment received	Medical	6 (54.5)	35 (81.4)	0.005	13.030	41 (75.9)
	Laser	1 (9.1)	6 (14)			7 (13)
	Surgical	1 (9.1)	2 (4.7)			3 (5.6)
	No treatment	3 (27.3)	0			3 (5.6)
Do you have a family history of glaucoma?	Yes	6 (7.4)	36 (53.7)	<0.001	55.651	42 (28.4)
	No	43 (53.1)	3 (4.5)			46 (31.1)
	Don’t know	32 (39.5)	28 (41.8)			60 (40.5)
If yes, mention the family with glaucoma diagnosis	Father	2 (33.3)	12 (33.3)	0.174	6.358	14 (33.3)
	Mother	1 (16.7)	19 (52.8)			20 (47.6)
	Sister	2 (33.3)	3 (8.3)			5 (11.9)
	Brother	1 (16.7)	1 (2.8)			2 (2.4)
	Grandfather	0	1 (2.8)			1 (2.4)

Table 3 evaluates participants’ knowledge of specific aspects of glaucoma. Urban participants consistently demonstrated greater knowledge than rural participants: 32.5% of urban and 11.9% of rural participants knew that glaucoma affects the optic nerve; 51.2% of urban versus 16.4% of rural participants knew that the risk of glaucoma increases with age. The statement “glaucoma is inherited” was known to 38.8% of urban participants and only 11.9% of rural participants. Additionally, 48.8% of urban participants knew that glaucoma is a progressive disease, compared to just 19.4% of rural participants. More urban participants knew that glaucoma is often asymptomatic (32.5% vs. 10.4%), linked to high IOP (52.5% vs. 22.4%), and can cause blindness (58.8% vs. 26.9%). Urban respondents were also more aware that glaucoma can be treated (57.5% vs. 31.3%) and were familiar with the available treatment modalities (drops, laser, surgery). Rural participants largely responded with “I don’t know,” highlighting significant knowledge gaps.

**Table 3:** Patients' knowledge-related questions Knowledge assessment was conducted only among participants who were aware of glaucoma. Among the 353 urban participants, 80 (22.7%) reported awareness, compared with 67 (19%) of the 353 rural participants.

Questions	Urban (n = 80), n (%)			Rural (n = 67), n (%)		
	Yes	No	I don’t know	Yes	No	I don’t know
Do you know that glaucoma affects the optic nerve?	26 (32.5)	22 (27.5)	32 (40)	8 (11.9)	34 (50.7)	25 (37.3)
The risk of glaucoma increases with age	41 (51.2)	17 (21.3)	22 (27.5)	11 (16.4)	2 (3)	54 (80.6)
Glaucoma is inherited	31 (38.8)	26 (32.5)	23 (28.7)	8 (11.9)	6 (9)	53 (79.1)
Glaucoma can occur without symptoms	26 (32.5)	28 (35)	26 (32.5)	7 (10.4)	6 (9)	54 (80.6)
Associated with high intraocular pressure	42 (52.5)	18 (22.5)	20 (25)	15 (22.4)	2 (3)	50 (74.6)
Glaucoma progresses over time	39 (48.8)	19 (23.8)	22 (27.5)	13 (19.4)	3 (4.5)	51 (76.1)
Glaucoma can be cured	38 (47.5)	19 (23.8)	23 (28.7)	12 (17.9)	4 (6)	51 (76.1)
Glaucoma has treatment	46 (57.5)	11 (13.8)	23 (28.7)	21 (31.3)	2 (3)	44 (65.7)
Do you know that glaucoma can be treated with drops, laser therapy, or surgery?	26 (32.5)	22 (27.5)	32 (40)	9 (13.4)	34 (50.7)	24 (35.8)
Glaucoma causes blindness	47 (58.8)	9 (11.3)	24 (30)	18 (26.9)	2 (3)	47 (70.1)

Table 4 summarizes overall glaucoma knowledge levels among urban and rural participants. A statistically significant association was found between the place of residence and knowledge level (p < 0.001). Excellent knowledge was more common among urban participants (31.3%) than rural participants (9%). Poor knowledge was predominant in rural areas (79.1%) compared to 51.2% in urban areas. Only 15% of all participants had “good” knowledge.

**Table 4:** Association between the knowledge level of glaucoma and the residence of the participants Values are expressed as frequency and percentage. The statistical relationship was assessed using the chi-square test.

Knowledge level	Urban (n = 80), n (%)	Rural (n = 67), n (%)	p-value	Chi-square value	Total, n (%)
Excellent (>75%)	25 (31.3)	6 (9)	<0.001	13.771	31 (21.1)
Good (50%-75%)	14 (17.5)	8 (11.9)			22 (15)
Poor (<50%)	41 (51.2)	53 (79.1)			94 (63.9)

## Discussion

Glaucoma is a leading cause of irreversible blindness, yet early diagnosis and timely treatment can effectively prevent vision loss. However, inadequate awareness and delayed presentation remain significant barriers to proper management and prevention of blindness associated with the disease. To the best of our knowledge, few comprehensive studies have simultaneously assessed glaucoma-related knowledge and awareness in both urban and rural populations of Bangladesh. In this cross-sectional study, we analyzed the level of knowledge and awareness regarding glaucoma among urban and rural populations, including a total of 706 respondents, with an equal distribution of 353 participants from each area.

The mean age of urban and rural respondents was not comparable (39.31 ± 14.1 vs. 52.01 ± 14.2 years). Female participants predominated in urban areas, whereas males were more common in rural areas. Interestingly, 53.7% of rural participants and only 7.4% of urban participants reported a family history of glaucoma. These findings are consistent with a study conducted in Bihar, India, by Sharma Pal [16].

Our study revealed that only 22.7% of urban and 19% of rural respondents were aware of the term “glaucoma.” Among those aware, 16.4% of urban and 18.1% of rural participants correctly identified glaucoma as an eye disease. Overall, respondents from urban areas demonstrated significantly higher awareness compared to their rural counterparts (Table 2). Notably, the overall level of awareness in our study was relatively low compared to previous studies in Bangladesh, where awareness levels ranged from 34.6% to 50% [15,19]. In contrast, a study in Ethiopia reported that only 2.4% of respondents were aware of glaucoma [20]. Similarly, studies conducted in various regions of India indicated lower levels of awareness among their study populations [21,22]. This variation in awareness levels across countries and regions may be attributed to differences in healthcare infrastructure, the reach and effectiveness of public health education campaigns, and the degree to which such programs penetrate urban versus rural communities.

Ocansey et al. reported that awareness about glaucoma in the general population is very poor [23]. In their study, over two-thirds of respondents were not aware of glaucoma, and more rural dwellers than urban dwellers were unaware of the condition. Even among those who reported being aware of glaucoma, some were unaware that it could cause blindness [23]. In our study, rural participants were more likely to have undergone an eye examination than their urban counterparts; however, urban respondents demonstrated greater awareness regarding key aspects of glaucoma. They were more likely to recognize it as a progressive disease, understand its potential to cause blindness, and be informed about treatment options. The reduced levels of awareness in certain populations may be attributed to lower educational attainment and less developed eye care systems, which limit access to both care and information.

Urban participants in our study consistently demonstrated a higher level of knowledge. For instance, 32.5% accurately identified the anatomical site affected by glaucoma, compared to only 11.9% of rural participants (Table 3). Similar patterns have been reported in studies by Sultana et al. and Becerril-Ledezma et al., who also highlighted greater knowledge among certain populations [15,24]. In Nepal, Sahu et al. reported that glaucoma awareness was only 14% among the rural population, compared to 65% in urban areas [25]. In Ghana, researchers found that overall knowledge of respondents about glaucoma was low [23]. Studies in India similarly revealed that knowledge of glaucoma among rural populations was very poor compared with urban populations [26]. These findings closely align with the results of our study.

A key finding of our study was the significant influence of education on glaucoma-related knowledge across both urban and rural groups. A marked disparity in educational attainment was observed, with 45% of rural respondents having no formal education. Participants with higher educational qualifications were statistically more likely to be aware of glaucoma, regardless of the place of residence. This association between education and awareness has also been supported by several studies conducted in northern and southern India, emphasizing that educational status plays a crucial role in improving knowledge and awareness of glaucoma [27,28].

In our study, the primary source of glaucoma awareness among rural participants was family members (47.8%), whereas urban participants most commonly cited relatives or friends (33.8%) and healthcare professionals (27.5%). In contrast, studies from southern India reported television and magazines as the most common sources of awareness, with family members affected by glaucoma ranking second. Similarly, surveys conducted in Germany found that friends were the leading source of glaucoma awareness (38%), surpassing physicians (16%), a trend also observed among urban populations in South India [26,29]. These differences highlight the varying impact of social networks and media exposure on health awareness across different cultural and geographic contexts.

Recall bias is an important limitation of this study, as it involved a degree of self-reporting. Participants were asked questions that required them to remember past events, which may have led to inaccuracies in their recollections, particularly among older individuals. To address the knowledge gap, strengthening community outreach, improving communication by healthcare providers, and integrating glaucoma awareness into routine eye care services are recommended. Enhancing public understanding is vital for facilitating early detection, ensuring timely treatment, and ultimately reducing the long-term burden of this preventable cause of blindness. These results can guide the development of targeted health education programs both within the study population and in similar communities, aiming to enhance awareness and knowledge of common eye diseases.

## Conclusions

This study highlights a significant disparity in glaucoma awareness and knowledge between urban and rural populations. Urban participants, with higher education and better access to private healthcare, showed greater awareness and understanding of glaucoma. In contrast, rural respondents, despite a higher prevalence of glaucoma and more frequent eye examinations, demonstrated poor knowledge, with many unaware that glaucoma is an eye disease or that it can lead to irreversible blindness. The findings suggest that lack of education and limited access to reliable information contribute to delayed diagnosis and poor disease management in rural areas. There is a clear need for targeted health education initiatives focusing on glaucoma, especially in underserved communities.

## Appendices

Questionnaire

ID No.:                                                                                                                   

Patient’s Socioeconomic Information

1. Gender

a. Male

b. Female

2. Age

_____ (years)

3. Marital status

a. Single

b. Married

c. Divorced/separated

d. Widowed

4. Area of residence   

a. Urban

b. Rural

5. Religion 

a. Muslim

b. Hindu

c. Others

6. Educational status (highest)

a. No formal education

b. Primary education

c. Secondary education

d. Higher secondary

e. Graduate

f. Post-graduate       

7. Monthly family income

a. <10,000

b. 10,000-20,000

c. 20,001-30,000

d. 30,001-40,000

e. 40,001-50,000

f. >50,000

8. Occupation     

a. Unemployed

b. Retired

c. Farmer

d. Business

e. Day labor

f. Service holder

g. Homemaker

h. Student

g. Others: __________________________

Awareness-Related Questions

1. Do you have history of eye examination?

a. Yes

b. No

If yes, the time of examination

a. Within 12 months

b. Before 12 months

If yes, place of eye examination

a. Government hospital/clinic

b. Private hospital/clinic

c. Outreach

2. Do you have history of hypertension?

a. Yes

b. No

c. Don’t know

3. Do you have a history of diabetes?

a. Yes

b. No

c. Don’t know

4. Have you ever heard of the term "glaucoma" before?

a. Yes

b. No 

If yes, please fill out the second page

5. Do you know that glaucoma is an eye disease?

a. Yes

b. No

c. Don’t know

6. Your source of information about glaucoma

a. Family member

b. Relatives/friends

c. Health professional

d. Mass media

7. Do you have a history of glaucoma?

a. Yes

b. No

c. Don’t know

If yes, types of treatment you received?

a. Medical

b. Laser

c. Surgical

8. Do you have a family history of glaucoma?

a. Yes

b. No

c. Don’t know

If yes, mention the family member with glaucoma diagnosis

__________________________

Knowledge-Related Questions (If Aware)

1. Altered anatomical site in glaucoma

a. Retina

b. Optic nerve

c. Cornea

d. I don’t know

2. Risk of glaucoma increases with age

a. Yes

b. No

c. I don’t know

3. Glaucoma is inherited

a. Yes

b. No

c. I don’t know

4. Glaucoma can occur without symptoms

a. Yes

b. No

c. I don’t know

5. Associated with high intraocular pressure

a. Yes

b. No

c. Not always

d. I don’t know 

6. Glaucoma progresses over time

a. Yes

b. No

c. I don’t know

7. Glaucoma can be cured

a. Yes

b. No

c. I don’t know

8. Glaucoma has treatment

a. Yes

b. No

c. I don’t know

9. Available treatment options

a. I don’t know

b. Only drops

c. Only surgery

d. Drops and surgery

e. Drops, laser, and surgery

10. Glaucoma causes blindness

a. Yes

b. No

c. I don’t know
